# Remyelination as a therapeutic target in the treatment of multiple sclerosis

**DOI:** 10.3934/Neuroscience.2025027

**Published:** 2025-11-06

**Authors:** Gurprit Kaur Garcha, Mohamed Ahmed

**Affiliations:** Department of Basic Science, College of Medicine, California Northstate University, 9700 West Taron Drive, Elk Grove, CA, 95757, USA

**Keywords:** neuroprotection, inflammation, oligodendrocytes, immune modulation, clinical trials

## Abstract

Multiple sclerosis (MS) is a chronic disease of the central nervous system (CNS) affecting young adults, particularly in North America and Europe, with nearly 2.5 million individuals impacted globally. Characterized by demyelination and neuronal damage, MS involves complex immune-mediated mechanisms. In this review, we focused on the pathophysiological processes of MS, highlighting the roles of T cells, B cells, and proinflammatory cytokines in driving demyelination, which are often the main focus of treatments in the form of immunotherapy. We emphasized remyelination as a key therapeutic target that is necessary for protecting axons and restoring neural function to solve the root problem. Emerging therapies, such as high-dose supplementation with vitamin D and glutathione, appear effective in regulating immune activity and lowering oxidative burden, thus supporting remyelination and neuroprotection. Preclinical models using toxin-induced demyelination have provided valuable insights into the mechanisms of remyelination and identified potential therapeutic targets like LINGO-1 antagonists. Clinical trials, particularly those involving the anti-LINGO-1 monoclonal antibody BIIB033, have demonstrated encouraging results in enhancing remyelination and improving clinical outcomes. LINGO-1 is an inhibitory protein that impairs OPC differentiation. Integrating these innovative approaches into clinical practice could revolutionize MS management by shifting the focus from managing symptoms to promoting CNS repair and long-term recovery. Continued research into the molecular mechanisms of remyelination and the development of targeted therapies is essential for advancing MS treatment and improving the quality of life for patients.

## 1. Introduction

MS is a multifactorial disease of the CNS of young adults with a high prevalence in North America and Europe, affecting almost 2.5 million individuals worldwide with an incidence rate that continues to rise [1]. During the course of the disease, some pathophysiological processes occur such as axonal damage, neuronal damage, demyelination, inflammation, gliosis, remyelination, and repair [2].

Remyelination has become a focal point in the search for novel therapeutic strategies aimed at repairing CNS damage in MS. In individuals with MS, white and gray matter are affected by lesions, which are characterized by the presence of demyelinated axons and a deficiency in functional myelin-producing oligodendrocytes (ODs) [3]–[6]. The inflammatory environment within these lesions is largely driven by an autoimmune attack against myelin, leading to sustained injury to ODs and neurons, which subsequently hampers the natural repair mechanisms of remyelination [7],[8].

A significant body of research supports the idea that T lymphocytes are the primary mediators of MS pathogenesis, as demonstrated in murine models and clinical studies [9]–[11]. However, B cells have also been implicated in disease progression [12]–[14]. These immune cells are capable of breaching the blood-brain barrier (BBB), entering the CNS, and mounting autoimmune responses against a variety of myelin and non-myelin antigens [7],[15]–[18]. This immune activity contributes to the formation of characteristic lesions visible via magnetic resonance imaging (MRI) [5],[19].

Within these active demyelinating sites, T cells, macrophages, and activated microglia release inflammatory mediators such as tumor necrosis factor-alpha (TNF-α) and nitric oxide, agents known to contribute significantly to demyelination and OD apoptosis in experimental settings [20]–[23]. As part of the cleanup process, macrophages engulf and remove myelin debris, which results in axonal regions devoid of insulation and impaired signal conduction [24].

Histologically, active lesions can be divided into zones: The core typically exhibits pronounced demyelination and macrophages loaded with myelin remnants, while the surrounding areas show variable degrees of myelin breakdown. The intermediate zone features macrophages containing myelin fragments but with reduced demyelination, and the outermost zone is distinguished by activated microglia and relatively intact myelin structures [25].

### 1.1. Understanding multiple sclerosis: Subtypes and therapeutic approaches

Based on the disease progression, there are several subtypes of MS [26]. Relapsing–remitting MS (RRMS) is characterized by periodic relapses of disease symptoms followed by a temporary remission [25]. Primary progressive MS (PPMS) is characterized by a continuous progression of illness with an absence of relapse or remission [25]. However, secondary progressive MS (SPMS) typically begins with several rounds of relapse and remission, eventually leading to an uninterrupted progression in disability [25].

Interestingly, in addition to targeting specific pathways or proteins in the differentiation of ODs, there are promising preventative measures that can be taken to prevent progression of MS or prevent it altogether. First, high vitamin D dose, in relation to modulating the immune system [27], and second glutathione, in relation to controlling oxidative stress [28], as discussed in later sections.

In this review, we focus on the processes of remyelination in response to demyelination in MS and several therapies designed to aid in the restoration of demyelinated axons. To better understand the processes of remyelination, we shed light on the processes of demyelination.

## 2. Demyelination in MS

### 2.1. History

The term myelin, referring to the insulating membrane surrounding axons, was introduced by German pathologist Rudolf Virchow [29]. In the CNS, this protective sheath is generated by specialized glial cells called ODs, which were first identified by Pío del Río Hortega in 1921 [30],[31].

### 2.2. Mechanism and pathology of demyelination

Demyelination, which may result from neurological disease or physical trauma, leads to the loss of the protective myelin sheath surrounding axons. This loss disrupts the efficient transmission of action potentials and leaves the exposed nerve fibers vulnerable to degeneration [32]–[34]. Action potential velocity can slow by >30 times its normal myelinated speed [35]. Demyelination causes diffuse distribution of channels, presumably as an attempt to maintain signal firing capabilities, albeit less efficiently [36]. Loss of myelin can result in a wide range of neurological disorders such as MS, including reduced motor function, impaired cognitive abilities, and vision problems [37]. The lesions in white matter and gray matter [38],[39] comprise demyelinated axons, with a lack of myelin-producing ODs [40],[41]. Lesional inflammation stems from an autoimmune reaction targeting myelin [42],[43], which can cause continuous damage to ODs and neurons and inhibit successful remyelination. Tissue damage seems to be driven by CD4+ Th1 cells which contribute to inflammation through the release of cytokines, whereas CD4+ Th2 cells are thought to play a regulatory role in counterbalancing these effects [44]. To study MS, experimental autoimmune encephalomyelitis (EAE) model is the most widely used animal models. It consists of an induced autoimmune disease in animals (typically mice or rats) and the induced disease mimics features of MS, including demyelination, neuroinflammation, paralysis, and T-cell infiltration into the CNS. Findings from EAE models, along with observations from immune responses and pathology in MS patients, indicate that the disease's immunopathology unfolds in several stages: (i) Priming of T cells, (ii) peripheral activation occurring in organs such as the thymus and lymph nodes, (iii) infiltration of pro-inflammatory T cells and monocytes into the CNS via the BBB, (iv) escalation of inflammation accompanied by activation of local antigen-presenting cells (APCs) like microglia, and (v) subsequent entry into the CNS parenchyma, leading to injury of ODs, the myelin sheath, and axons [45].

Within secondary lymphoid organs, APCs display myelin-related antigens that trigger the activation and proliferation of T cells specifically reactive to myelin. Once activated, these T cells circulate throughout the body in search of their target antigens to initiate a secondary immune response upon re-encounter [46]. This process leads to their migration across the BBB, which occurs via interactions between adhesion molecules found on the surface of lymphocytes and endothelial cells [47]. The migration process is a multi-step process that starts with the slow movement of circulating T cells in the bloodstream due to an interaction between distinct adhesion molecules on their surface and on endothelial cells [44]. A key component of this cascade is the integrin very late antigen-4 (VLA-4), expressed on T cells, which binds to vascular cell adhesion molecule-1 (VCAM-1) on endothelial cells to enable firm adhesion and diapedesis into the CNS. This VLA-4/VCAM-1 interaction is critical for T cell entry and has been effectively targeted by therapeutics like natalizumab [48]. During the second phase of immune activation, certain chemokines, particularly CCL19 and CCL21, are secreted to support T cell activation [49]. The subsequent third and fourth stages involve the firm adhesion of lymphocytes to the vascular endothelium and their passage through the BBB, facilitated by adhesion molecules like ICAM-1 and VCAM-1 [44]. In the fifth phase, CD4+ T cells recognize their specific antigens, such as components of myelin, presented by major histocompatibility complex (MHC) class II or CD1 molecules on APCs like perivascular dendritic cells [50]. This antigen recognition at the immune synapse leads to reactivation of the T cells [44].

After crossing the BBB, these autoreactive CD4+ T cells trigger a cascade of local inflammatory responses [44]. One of the defining pathological outcomes of this process is the formation of demyelinated plaques, accompanied by the activation of astrocytes, a phenomenon known as gliosis [51].

### 2.3. Myelin destruction

In MS, demyelination is linked to the disruption of the structural organization within the paranodal and juxtaparanodal regions of axons [44]. In areas where myelin has been lost, proteins typically confined to these regions become irregularly dispersed along the exposed axonal surfaces [52]. One of the early pathological signs includes the abnormal co-localization of Neurofascin (Nf)-155-positive paranodal sites with juxtanodal Kv1.2 channels, particularly near actively demyelinating white matter lesions that exhibit axonal damage [53].

## 3. Remyelination

Remyelination is considered the step immediately prior to the repair process throughout the course of MS. Remyelination is a process that involves multiple steps and is triggered in response to demyelination [54].

### 3.1. Mechanism of remyelination

**Figure 1.**
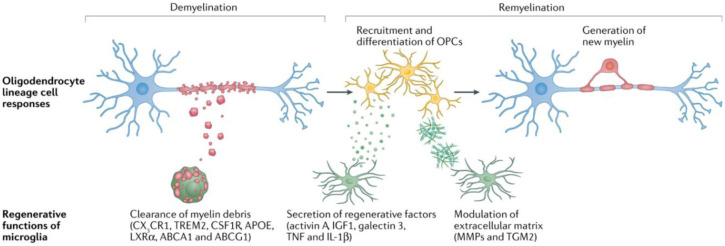
Illustration of the demyelination process initiating a cascade of restorative signaling factors that promote remyelination, a fundamental mechanism in neural repair [62].

Remyelination is the generation of new myelin sheaths around denuded axons in the adult CNS [55]. Proper redistribution of ion channels at the nodes of Ranvier and restoration of saltatory conduction is considered an immediate consequence of remyelination [56],[57]. More importantly, evidence suggests that demyelinated axons are better protected from subsequent injury when they become remyelinated, perhaps by restoring proper growth factor signaling between the OD and the axon [39],[58]–[61]. The way in which the microglia clear the myelin debris, secrete regenerative factors, and modulate the extracellular matrix to support OPC recruitment, differentiation, and the eventual generation of new myelin is an immensely coordinated process of demyelination and remyelination (Figure 1) [62].

Pepinsky et al. evaluated the CNS exposure and efficacy of the anti-LINGO-1 Li81 antibody following systemic administration in rodent models of spinal cord remyelination [63]. Concentration levels of Li81 were measured in spinal cord, brain tissue, and cerebrospinal fluid (CSF), and these levels were compared to remyelination outcomes in both lysolecithin-induced and EAE demyelination models [63]. The results demonstrated a dose-dependent response, where increased levels of Li81 in spinal cord tissue were closely associated with improved myelin repair. These effects aligned with the known binding affinity of the antibody for the LINGO-1 protein [63].

Pharmacokinetic analysis showed that while serum and CNS drug concentration profiles mirrored each other, only a limited portion of the injected compound penetrated the CNS [63]. CNS drug levels, expressed as a percentage of the corresponding blood levels, rose slightly over time, likely due to the progressive accumulation of the antibody at its target site [63]. This effect was most noticeable when systemic concentrations decreased, suggesting that even a small amount of the drug could engage with its CNS target. However, the overarching obstacle in neurotherapeutics remains the BBB, which functions as a formidable barrier via tightly regulated endothelial junctions and limited cellular transport mechanisms [64].

In addition to Li81, another antibody, rHIgM22, a human-derived IgM, has also shown promise in encouraging remyelination in experimental MS models [65],[66]. Although the precise mechanism behind its effect remains elusive, some findings propose that rHIgM22 may support the survival of immature ODs by promoting anti-apoptotic pathways, as well as reducing caspase-3 activity and related gene expression [67],[68].

### 3.2. Migration and differentiation

Oligodendrocyte progenitor cells (OPCs) are derived from neural stem cells through specific induction signals [69],[70]. These progenitors have the ability to multiply and migrate toward areas of demyelination within the CNS [71]. Various growth factors, including hepatocyte growth factor, basic fibroblast growth factor, and vascular endothelial growth factor A, play critical roles in guiding both the migration and differentiation of these precursor cells in vitro [72],[73]. As OPCs mature, they progress through distinct developmental stages. Early-stage cells can be identified by markers such as the A2B5 ganglioside [74]–[76] and the NG2 proteoglycan [77],[78]. The transition to the pre-OD phase is marked by expression of the O4 sulfatide [79],[80]. Fully differentiated ODs represent the final stage of this process [54]. These mature cells are responsible for producing myelin and are characterized by the presence of key myelin-associated proteins, including proteolipid protein (PLP) [81],[82], myelin basic protein (MBP) [83], myelin-associated glycoprotein (MAG) [84],[85], and myelin OD glycoprotein (MOG) [86],[87]. One mature OD has the capacity to myelinate several axons simultaneously [88]. However, if these cells fail to successfully connect with and ensheath axons, they are typically eliminated through programmed cell death, emphasizing the need for precise targeting during OD migration [89],[90].

### 3.3. Myelin production

Mature ODs are responsible for forming the myelin sheath that envelops and insulates neuronal axons [91]. This sheath is composed primarily of lipids, with proteins making up approximately 30% of its total structure [92]. Among the key membrane proteins, MAG is essential for facilitating interaction between myelin and axons [93], while PLP and MBP are believed to play structural roles in maintaining the integrity of the sheath [94]. The initiation of remyelination occurs once ODs reach full maturity and is further influenced by components of the extracellular matrix [95].

During the remyelination process, newly synthesized myelin layers gradually wrap around demyelinated axons until restoration is complete [96]. Interestingly, many of the proteins involved in forming the new sheath are synthesized locally at the site of remyelination than in the OD's main cell body [96]. This is made possible by a network of microtubules that transport ribosomes and messenger RNA (mRNA) from the OD body directly to the remyelination site [63],[97],[98].

## 4. Treatment options for multiple sclerosis

The treatment strategies for MS largely focus on modulating or suppressing the immune system to reduce relapse rates, delay disease progression, and control the formation of new lesions [99]. These approaches are effective to varying degrees, especially in the early stages of the disease, but they do not reverse existing damage or fully halt the disease process.

There are now over twenty approved disease-modifying therapies (DMTs) for MS. These include injectable medications such as interferon beta and glatiramer acetate, oral agents like fingolimod, dimethyl fumarate, and teriflunomide, and monoclonal antibody treatments, including natalizumab, ocrelizumab, and alemtuzumab [100]. These therapies work primarily by suppressing or redirecting immune activity and are particularly effective in reducing relapse rates in RRMS, often by 30 to 70 percent depending on the drug [101]. However, their efficacy diminishes significantly in progressive forms of MS, where inflammation is less prominent, and neurodegeneration dominates the clinical picture [101].

Despite their benefits, current DMTs come with limitations. They do not promote remyelination or restore neurological function already lost. Moreover, many have significant side effects, including immunosuppression, liver toxicity, cardiovascular risks, and a heightened risk of infections such as progressive multifocal leukoencephalopathy (PML), especially with drugs like natalizumab [102]. These risks complicate long-term treatment adherence and can lead to reduced quality of life for patients who are already coping with a complex chronic illness.

Symptomatic management is another important aspect of MS care and includes interventions like physical therapy, muscle relaxants, bladder control agents, antidepressants, and treatments for fatigue and pain. While these measures can improve daily functioning and comfort, they do not affect the disease course itself [103]. MS continues to significantly impair quality of life due to cumulative physical disability, cognitive decline, and the emotional toll of a progressive neurological condition. Many patients, particularly those with SPMS or PPMS, continue to experience worsening symptoms even when on DMTs [103].

The central challenge in MS treatment today is the lack of therapies that address the core problem of demyelination and axonal injury [104],[105]. Present medications predominantly target inflammation without repairing the existing damage or halting the neurodegenerative process [102],[106]. This creates an urgent need for treatments that can restore myelin, rebuild neuronal pathways, and preserve long-term CNS function [107],[108].

Remyelination represents a promising therapeutic avenue that directly addresses this need. Unlike current approaches, which suppress the immune system, remyelination therapies aim to regenerate damaged myelin and restore electrical conduction along neurons. This regenerative capacity is critical because remyelination not only reinstates functional signal transmission but also protects axons from irreversible degeneration, a major contributor to permanent disability in MS [104]. Although spontaneous remyelination can occur in early disease stages, this process often fails over time due to age, chronic inflammation, and changes in the lesion microenvironment.

Several investigational therapies are focused on promoting remyelination. Clemastine fumarate, an antihistamine that acts as an antimuscarinic agent, has demonstrated efficacy in promoting remyelination in the optic nerve and improving conduction latency in patients with optic neuritis [109]. Similarly, the anti-LINGO-1 monoclonal antibody opicinumab has been studied in multiple trials, including RENEW, SYNERGY, and AFFINITY. While the AFFINITY trial was discontinued due to insufficient clinical benefit, these studies have paved the way for understanding remyelination pathways and how they might be therapeutically targeted [110]. These trials have also underscored the complexity of remyelination and the need for better biomarkers and patient selection to enhance treatment responses.

Additionally, research into adjunctive agents such as vitamin D and glutathione (GSH) suggests these compounds may indirectly support remyelination by improving the inflammatory and oxidative environment of MS lesions [111],[112]. Although these agents are not direct remyelinating drugs, their role in modulating immune responses and protecting OPCs supports a comprehensive, multi-targeted treatment strategy.

Remyelination therapies hold the promise not just of symptom management or delayed progression, but of true functional restoration [107]. For a disease that lacks a cure and leaves many patients with accumulating disability, such a shift in therapeutic strategy would be transformative [113]. Developing therapies that successfully promote remyelination would not only address a fundamental pathological feature of MS but also significantly improve long-term outcomes and quality of life for patients [108],[114].

## 5. Therapeutic potential of vitamin d and glutathione

Vitamin D and GSH have emerged as compelling therapeutic candidates in the context of remyelination in MS. While traditionally studied for their roles in immunomodulation and antioxidant defense, mounting evidence from both clinical and animal studies now supports their involvement in facilitating remyelination [111],[115],[116]. These agents may help modify the disease course not only by reducing inflammation but by improving the microenvironment necessary for OPC proliferation, survival, and maturation [117].

Vitamin D exerts its effects via the vitamin D receptor (VDR), expressed in multiple immune and CNS cell types. Its immunomodulatory role includes enhancing regulatory T cell (Treg) differentiation and suppressing Th1 and Th17 responses, thus decreasing pro-inflammatory cytokines such as IFN-γ and IL-17 [118]–[120]. Low serum levels of vitamin D have been associated with increased MS prevalence, particularly in regions with limited sunlight exposure [121]. Higher serum concentrations are also correlated with reduced relapse rates and slower disease progression [121].

Importantly, several studies have shown that vitamin D can promote remyelination. In the cuprizone model, a well-established demyelination model, vitamin D supplementation has been found to accelerate remyelination and increase OD lineage cell density [115]. Similarly, in the EAE model, vitamin D reduces inflammation, preserves myelin integrity, and supports OD survival [122]. Additionally, vitamin D modulates cholesterol and oxysterol metabolism, which are critical for myelin membrane synthesis, further enhancing its relevance in demyelination/remyelination models [117]. These findings underscore its dual function, both anti-inflammatory and regenerative, in MS pathology.

Vitamin D also demonstrates synergistic potential with MS therapies. High-dose supplementation (e.g., 5000 IU/day) has been studied as an adjunct strategy, with some trials suggesting it may reduce relapse frequency and enhance the efficacy of DMTs [121],[123]. Moreover, vitamin D directly affects OPCs and ODs through VDR signaling, promoting their proliferation and differentiation, which are key processes for remyelination [116].

GSH, a major endogenous antioxidant, plays an equally vital role in MS by countering oxidative stress, contributing to demyelination and axonal injury [124],[125]. GSH detoxifies reactive oxygen species (ROS) and preserves mitochondrial function, both of which are often disrupted in MS [124],[126]. In the EAE model, boosting GSH levels or activating the Nrf2 pathway reduces neuroinflammation and partially preserves myelin [111]. Although direct evidence linking GSH to enhanced remyelination is limited, its role in protecting the lesion microenvironment and supporting OD survival makes it a promising therapeutic adjunct.

Notably, GSH and vitamin D also modulate immune responses. GSH may regulate T cell function and BBB integrity, while vitamin D influences both innate and adaptive immune cells, including macrophages, neutrophils, and B cells [127]–[129]. The overlapping immunological and neuroprotective actions of these compounds make them promising combination agents for future remyelination-focused therapies.

While no direct mechanistic link has been established between vitamin D, GSH, and LINGO-1, their effects may complement anti-LINGO-1 strategies. Creating a microenvironment with reduced oxidative stress and inflammation, achievable through vitamin D and GSH, could enhance the effectiveness of anti-LINGO-1 therapies in promoting remyelination [111],[130].

**Figure 2.**
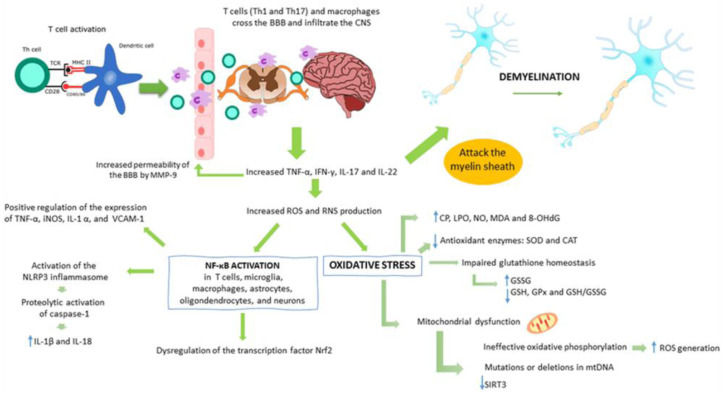
This image illustrates the role of neuroinflammation, oxidative stress, and mitochondrial dysfunction in promoting demyelination through T cell infiltration, cytokine release, and ROS production in MS [131].

In summary, vitamin D and GSH contribute to remyelination not only through immunomodulation but also via mechanisms that support OPC function, myelin repair, and cellular resilience under inflammatory stress [116],[117],[122]. These agents offer a unique therapeutic angle, one that may enhance the efficacy of other remyelination-promoting approaches and therefore merit focused investigation in MS research and clinical trials. Moreover, the interplay of neuroinflammation, oxidative stress, and mitochondrial dysfunction has been shown to exacerbate demyelination by promoting T-cell infiltration, pro-inflammatory cytokine release, and excessive reactive oxygen species (ROS) production within the central nervous system (Figure 2) [131].

## 6. Preclinical studies

Because enhancing remyelination is deemed crucial for neuroprotection in MS and therefore a major line of defense against progressive disease, toxin-induced MS models are among the most significant tools for translational research [132]. Two models are used extensively. For one, the copper chelator cuprizone (0.2% in chow) is fed to mice of a susceptible strain for 4–6 weeks. Cuprizone causes dysfunction of mitochondrial complex IV, with selective toxicity for ODs among CNS cells [133]–[135]. ODs in the corpus callosum and hippocampus of cuprizone-fed mice undergo apoptosis after 3 weeks of treatment [136]. After cuprizone is discontinued, remyelination ensues [39],[133]. This model provides insights into the determinants of OD cell death [137]–[139]. More extensively, the model has been used to examine mechanisms of remyelination, with frequently surprising results [140]. The lack of TNF-α led to a significant delay in remyelination as assessed by histology, immunohistochemistry for myelin proteins and electron microscopy coupled with morphometric analysis [140]. Investigating the mice lacking TNF receptor 1 (TNFR1) or TNFR2 indicated that TNFR2, not TNFR1, is critical to OD regeneration [140]. A primary target of demyelination in the cuprizone model is the corpus callosum [141],[142]. Demyelination occurred after 3 weeks of cuprizone administration and progressed to completion by 5 weeks [141],[143]. Removal of cuprizone at 6 weeks led to remyelination one week later [144]–[146]. This unexpected reparative role for TNF-α in the CNS is important for understanding OD regeneration/proliferation, nerve remyelination and may be a good target of new therapeutics for demyelinating diseases [144]–[146].

Hall was the first to use the detergent lysophosphatidylcholine, the membrane-dissolving agent, as a demyelinating agent [147]. It has since been used in numerous studies of mice, rats, rabbits, and cats. It is normally used as a 1% saline solution [148]. Injection-based toxic models have been used as well to identify strategies to enhance remyelination. Microinjection of the detergent lysophosphatidylcholine (or ethidium bromide, which is now used less frequently) into the spinal cord white matter tracts causes prompt demyelination, followed by remyelination [133],[149]. This model has been used very productively to examine cellular and molecular determinants of remyelination [150],[151]. This procedure produces a well characterized demyelinating injury consists principally of macrophage/microglial infiltration and activation [152], reactive astrogliosis, perturbation of axonal homeostasis/axonal injury, and OPC proliferation and migration [153]. The lesion predictably evolves over the period of a few weeks and is capable of full remyelination. This method has been particularly useful in studying the choreography of events involved in de- and remyelination. Further, it has been adopted as a tool for pre-clinical testing of candidate therapies to accelerate repair following a demyelinating insult [154].

Pavelko et al. hypothesized that spontaneous remyelination in the toxic non-immune model of lysolecithin-induced demyelination is enhanced by manipulating the inflammatory response [155]. In PBS- treated SJL/J mice, the number of remyelinating axons/ mm2 of lesion area increased significantly 3 and 5 weeks after lysolecithin injection in the spinal cord. After 3 weeks, the number of remyelinating axons in the lysolecithin treatment groups was similar to the spontaneous remyelination in the 5 weeks PBS control-treated group, indicating that these treatments promoted remyelination [155].

## 7. Possible causes of remyelination failure

Remyelination involves complex molecular interactions between OPCs, axons, astrocytes, microglia, and macrophages, which coordinate to restore lost myelin sheaths following injury [107],[156]. The extent of remyelination in MS patients is variable and tends to decrease with age and disease progression [157],[158]. Early in the disease, episodes of demyelination are often followed by partial remyelination, which contributes to clinical recovery after relapse [159],[160]. However, as the disease progresses, remyelination becomes largely insufficient, potentially due to an imbalance between regenerative signals and persistent inhibitory factors [113].

One major reason for remyelination failure is the impaired recruitment, migration, or differentiation of OPCs at the lesion site [161]. In some cases, OPCs are present in demyelinated lesions but fail to differentiate into mature, myelinating ODs due to a hostile microenvironment [162]. Factors such as chronic inflammation, disrupted extracellular matrix, and the presence of reactive astrocytes can inhibit OPC maturation [163],[164]. Reactive astrocytes, in particular, release molecules like chondroitin sulfate proteoglycans (CSPGs), which form a physical and biochemical barrier to OPC migration and differentiation [165].

Another contributing factor is the prolonged presence of pro-inflammatory cytokines, such as TNF-α, IL-1β, and IFN-γ, which can create a toxic environment for ODs and promote demyelination over remyelination [166]. In contrast, anti-inflammatory microglia and macrophages are known to support remyelination by clearing debris and releasing growth factors, but their activation states can shift toward a more detrimental phenotype in chronic MS lesions [167]. Moreover, myelin debris can persist in MS lesions due to inefficient clearance mechanisms, thereby inhibiting OPC differentiation and remyelination [168].

Epigenetic and metabolic changes in OPCs are also implicated in remyelination failure. Aging leads to reduced histone acetylation and mitochondrial dysfunction in OPCs, diminishing their regenerative capacity [158],[169]. Additionally, insufficient cholesterol availability and altered lipid metabolism within demyelinated lesions can impair myelin membrane synthesis [170]. Genetic predisposition may further modulate remyelination capacity, with variants in genes like LINGO1 shown to inhibit OPC differentiation and myelination [171].

The cause of remyelination failure may also vary between lesions within the same patient due to heterogeneity in immune activity, lesion age, and the degree of axonal preservation [172],[173]. In some lesions, axonal damage is so extensive that even successful remyelination would be insufficient to restore function [108]. Therefore, a deeper understanding of lesion-specific factors and molecular pathways that regulate remyelination is essential for developing effective repair strategies in MS.

## 8. The concept of remyelination as a therapeutic target for treating MS

In the CNS, axons are wrapped in a myelin sheath that enables the rapid and efficient conduction of electrical signals throughout the body [174]. This insulating layer is produced by ODs, the specialized glial cells responsible for generating myelin. In MS, white and gray matter are affected, and the disease's dual inflammatory and degenerative mechanisms lead to the progressive loss of myelin, ODs, neurons, and their axons [174]. A hallmark of MS pathology is the ongoing disruption of the OD-myelin unit, which persists throughout the course of the disease [174]. As demyelination continues unchecked, it outpaces the body's natural ability to remyelinate. Restoring myelin could protect vulnerable neurons and axons from further damage, making remyelination a critical focus of current research. Despite this, no MS therapies currently approved by the FDA have succeeded in effectively promoting remyelination [175].

One promising molecular target in this context is LINGO-1, a transmembrane protein found specifically on neurons and ODs [175]. This protein acts as a negative regulator of both myelination and OD maturation [176] and plays a role in inhibiting the repair of injured axons [154].

**Figure 3.**
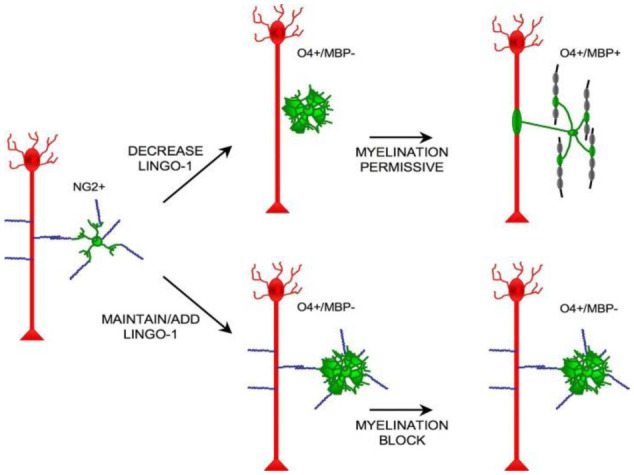
Illustration depicting the impact of Lingo-1 protein on myelination, highlighting its regulatory role in inhibiting or modulating the remyelination process [178].

LINGO-1 is part of a complex with other proteins such as Nogo receptor 1 (NgR1) and p75 which binds to myelin-associated inhibitors [177]. This binding activates intracellular signaling pathways that lead to the inhibition of axon regeneration [177]. Additionally, LINGO-1 inhibits the differentiation of ODs by binding to specific receptors on OD precursor cells (Figure 3) [178]. Pepinsky et al also indicated that LINGO-1 antagonist antibodies are currently being investigated as a way to restore remyelination as a new paradigm for treatment of individuals with MS [176].

The anti-LINGO-1 Li81 antibody, BIIB033, is currently in clinical trials and is the first MS treatment targeting CNS repair, according to Pepinsky et al [176].

### 8.1. Randomized phase I trials of the safety/ tolerability of anti-LINGO-1 monoclonal antibody BIIB033

**Figure 4.**
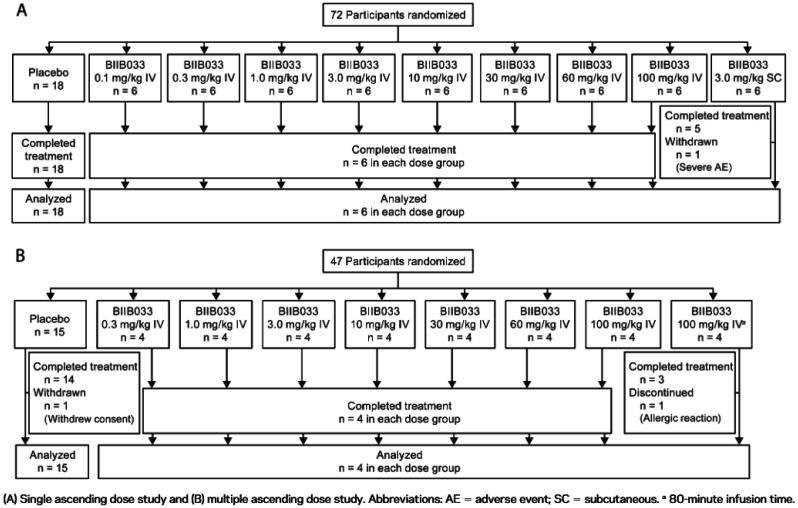
Overview of treatments (derived) [179].

The purpose of this study was to assess the safety profile, tolerability, and pharmacokinetics (PK) of BIIB033, an anti-LINGO-1 monoclonal antibody, in healthy individuals and patients diagnosed with MS. Researchers conducted two separate randomized, placebo-controlled trials. In the first, single ascending doses (SAD) ranging from 0.1 to 100 mg/kg were delivered either intravenously or subcutaneously to 72 healthy participants [179]. The second phase involved multiple ascending doses (MAD), with participants receiving two intravenous (IV) infusions of BIIB033 (0.3–100 mg/kg), spaced 14 days apart. This phase included 47 individuals with either relapsing-remitting or SPMS [179]. Safety monitoring encompassed adverse event tracking, neurological assessments, conventional and advanced MRI techniques, EEG, optical coherence tomography, retinal imaging, and evoked potential tests. Additionally, serum and CSF pharmacokinetics, along with the immunogenic response to BIIB033, were analyzed [179] (Figure 4).

### 8.2. BIIB033

BIIB033 is a human-derived, aglycosylated IgG1 monoclonal antibody designed to target LINGO-1 with strong specificity and binding affinity [180]. It is under investigation as a potential therapeutic aimed at promoting remyelination and providing protection or repair for damaged axons in individuals with MS [172]. Administering BIIB033 at high systemic doses, combined with the fact that LINGO-1 is selectively expressed in the CNS, may help achieve therapeutic concentrations within the brain and spinal cord, offering a possible solution to the challenge of limited antibody penetration across the BBB [63].

### 8.3. The outcome of the study

The studies assessing BIIB033 demonstrated that single and double doses, up to 100 mg/kg, were generally well tolerated in healthy individuals as well as in MS patients [179]. No significant adverse events were observed, with tolerability appearing consistent across both groups [179]. Pharmacokinetic analysis revealed that serum profiles of BIIB033 were comparable between healthy volunteers and those with MS, characterized by a low volume of distribution (VD), suggesting minimal tissue binding within the distribution range [179]. This limited VD aligns with the pharmacological behavior commonly observed in other IgG-class antibodies [181],[182]. Additionally, the ratio of BIIB033 concentrations in CSF relative to serum was approximately 0.1%–1%, which is in line with reported values for other IgG monoclonal antibodies [179].

Notably, CSF concentrations of the anti-LINGO-1 monoclonal antibody required to achieve 50% and 90% of the maximal remyelination effect in the spinal cord were estimated at approximately 30 ng/mL and 100 ng/mL, respectively (EC50 and EC90) [179]. Based on an assumed CSF penetration of 0.1%, these values would correspond to serum concentrations of roughly 30 mg/mL for EC50 and 100 mg/mL for EC90 [179]. In clinical participants with MS, IV doses as low as 0.3 mg/kg and 0.10 mg/kg resulted in average BIIB033 serum levels that exceeded both EC50 and EC90 thresholds, indicating the potential for pharmacologically meaningful effects at these doses [179].

Collectively, data from the two phase I clinical trials demonstrated that BIIB033 was well tolerated at doses up to 100 mg/kg, with minimal signs of immunogenicity [179]. The ability of higher doses to achieve therapeutic serum concentrations further supports the progression of BIIB033 into phase II trials focused on proof-of-concept and clinical efficacy [179].

### 8.4. RENEW Study [183]: Evidence of remyelination following anti-LINGO-1 therapy (LINGO-BIIB033), a monoclonal antibody targeting LINGO-1, administered after acute optic neuritis (AAN 67th Annual Meeting Abstract)

RENEW [183] was a randomized, double-blinded, placebo-controlled, parallel-group, phase II study. The researchers aimed to determine the efficacy of the anti-LINGO-1 antibody BIIB033 for CNS remyelination in subjects with first unilateral acute optic neuritis (AON) episode [184]. A unilateral AON episode refers to a sudden onset of inflammation of the optic nerve in one eye [184]. The exact cause of optic neuritis is often unknown, but it can be associated with autoimmune diseases like MS [185]. This study was the first to combine functional, structural, and clinical efficacy endpoints in AON [184].

Inclusion criteria involved subjects (18 to 50 years) who completed treatment with high-dose steroids and were then randomized 1:1 to 100 mg/kg BIIB033 IV or placebo once every four weeks and followed up to Week 32. Eighty-two subjects across 33 sites in Europe, Canada and Australia received a total of 6 IV infusions of 100 mg/kg BIIB033. Remyelination was evaluated by recovery of optic nerve conduction latency using full-field visual evoked potential (FF-VEP) compared with the unaffected fellow eye at baseline [184]. Neuroprotection was studied by measuring the thickness of the retinal nerve fiber layer (RNFL) and ganglion cell layer using spectral-domain optical coherence tomography (SD-OCT) and change in low-contrast letter acuity (LCLA). ANCOVA and MMRM in the per-protocol (PP) and intent-to-treat (ITT) populations were used for between-treatment comparisons. RENEW is the first clinical trial reporting on the efficacy of BIIB033. The observed shortening of the FF-VEP provides evidence of proof of biology for remyelination [184].

The researchers for the “RENEWED: A follow-subsequent analysis of the Phase II RENEW trial evaluating opicinumab in acute optic neuritis” aimed to evaluate the long-term effects of opicinumab, an anti-LINGO-1 monoclonal antibody, as a potential remyelinating therapy for AON, a condition frequently associated with MS [184]. The original RENEW study [183] was a randomized, placebo-controlled phase 2 trial that investigated the efficacy and safety of opicinumab in patients experiencing their first episode of unilateral AON. While no significant improvement in visual evoked potential (VEP) latency recovery was observed in the ITT population, the per-protocol (PP) population exhibited a greater degree of remyelination with opicinumab compared to placebo [184]. To further assess long-term electrophysiological and clinical outcomes, the RENEWED follow-up study [184] was conducted two years after the last RENEW study visit, examining changes in full-field VEP (FF-VEP) latency, multifocal VEP (mfVEP) latency, and the progression to clinically definite MS (CDMS) [184].

The researchers enrolled 52 of the original 82 RENEW participants, with 28 from the opicinumab group and 24 from the placebo group [184]. The primary endpoint assessed FF-VEP latency changes in the affected eye compared to the fellow eye at baseline in the original RENEW study. While numerical reductions in latency delay were observed with opicinumab, the differences did not reach statistical significance in either the PP or ITT populations [184]. The adjusted mean difference in FF-VEP latency delay between the opicinumab and placebo groups was −6.0 ms (p = 0.165) in the PP population and −4.5 ms (p = 0.274) in the ITT population, indicating a potential but inconclusive effect of opicinumab on remyelination [184]. However, in the subset of participants included in the mfVEP substudy, a significant reduction in latency delay was observed in the opicinumab group compared to placebo, with an adjusted mean difference of −19.6 ms (p = 0.009) in the PP population and −14.0 ms (p = 0.038) in the ITT population [184]. This suggests that mfVEP may be a more sensitive measure of remyelination effects than FF-VEP.

The researchers also evaluated the incidence of CDMS in participants who had not been diagnosed at the baseline of the RENEW study [184]. In the PP population, 55% of participants in the opicinumab group developed CDMS compared to 67% in the placebo group, with an estimated proportion of 0.50 versus 0.61, respectively [184]. The median time to CDMS diagnosis was longer in the opicinumab group (909.5 days) compared to the placebo group (386.0 days), but the difference was not statistically significant (hazard ratio 0.60, p = 0.23) [184]. No significant differences were observed in visual acuity, RNFL thickness, or expanded disability status scale (EDSS) scores between the treatment groups, suggesting that opicinumab did not provide a measurable benefit in structural or functional vision outcomes [184].

The implications of this study for remyelination as a treatment for MS are significant. While the observed reductions in VEP latency delay, particularly in the mfVEP substudy, suggest that opicinumab may facilitate remyelination, the overall results were not conclusive due to the small sample size and study limitations [184]. The findings reinforce the challenges of promoting effective remyelination in MS and highlight the need for early intervention, as prolonged demyelination can lead to irreversible axonal damage [184]. Additionally, the study underscores the importance of identifying more reliable biomarkers for remyelination, as the discrepancies between FF-VEP and mfVEP results indicate that different assessment techniques may yield varying sensitivity to changes in myelin repair [184]. Although opicinumab did not demonstrate a statistically significant effect on remyelination in this study, the potential delay in CDMS onset and improvements in mfVEP latency suggest that further investigation into LINGO-1 inhibition as a therapeutic strategy for MS may be warranted [184]. Future studies with larger sample sizes and optimized methodologies will be necessary to determine whether opicinumab or similar therapies can provide clinically meaningful benefits for individuals with MS [184].

### 8.5. SYNERGY Study [186]: Phase II trial evaluating BIIB033's efficacy, safety, tolerability, and PK in relapsing MS patients receiving Avonex

The primary objective of the study was to evaluate the efficacy of BIIB033 in participants with active relapsing MS when used concurrently with Avonex (interferon β-1a) [186]. The secondary objectives of this study population were to assess the safety, tolerability, and population PK of BIIB033 when used concurrently with Avonex [186]. A total of 419 participants were involved in the study across 70 sites in the USA, Canada and Europe [186]. Inclusion criteria involved participants who were diagnosed with RRMS or onset of Secondary Progressive Multiple Sclerosis (SPMS) [186]. RRMS and SPMS subjects must have evidence of ongoing disease activity within 12 months of enrollment [186]. A total of 331 subjects had RRMS and 88 had SPMS. Also, all male and female subjects of childbearing potential must practice effective contraception during the study and be willing and able to continue contraception for at least 6 months after their last dose of study treatment [186]. The majority of subjects were females and white. The mean age of all subjects was 40 years, and those with SPMS were older (mean age = 46 years). Most subjects had 2 or more relapses within the past 2 years (69%) [186].

However, candidates with the following criteria were excluded from the study: a) Patients with MS relapse that has occurred within the 90 days prior to Day 1/Baseline and/or the subject has not stabilized from a previous relapse prior to screening previous history of clinically significant disease. b) Patients planning to undergo elective major procedures/surgeries at any time during the study. c) Patients treated with any investigational MS drugs within 3 weeks or 5 times the half-life (whichever is longer) prior to Day1/Baseline. d) RRMS subjects with any history of inadequate response to any approved interferon β preparation. e) Patients with a history of HIV, HCV antibody, or HBV. f) Patients with a history or evidence of drug or alcohol abuse within 2 years prior to randomization [186]. Four hundred and nineteen participants were included in the study according to the above outlined inclusion and exclusion criteria. There was no gender restriction for the involvement in the study. Participants were assigned as 3 intervention groups vs. a placebo group. The intervention groups were treated as following: Group I (BIIB033, 3 mg/kg once every 4 weeks IV Infusion), group II (BIIB033, 10 mg/kg BIIB033 10 mg/administered intravenously every four weeks at a dose of X kg), group III (BIIB033, 30 mg/kg BIIB033 30 mg/administered intravenously every four weeks at a dose of X kg), and group IV (100 mg/kg BIIB033 100 mg/administered intravenously every four weeks at a dose of X kg). Intervention groups were tested against placebo (once every 4 weeks IV infusion). All participants, including placebo, received an IM injection of IFNβ-1a (19%), glatiramer acetate (18%), IFNβ-1b (9%), natalizumab (7%), and mitoxantrone (6%) once a week. The starting date of the study was August 2013, and the estimated study completion date was March 2016 [186]. The primary endpoints being studied were cognitive function with the secondary endpoint being neuro-physical outcomes. At the end of treatment (after 72 weeks, approximately 18 months), the treatment groups had increased cognitive function and lower rates of neurophysical decline.

The study “mapping progressive demyelination in gradually enlarging MS lesions/Evolving White Matter MS Lesions” investigated the role of slowly expanding/evolving lesions (SELs) in MS and their impact on disease progression. SELs are chronic lesions that continue to expand over time, contributing to progressive disability through persistent demyelination and axonal loss [187]. The researchers utilized data from the SYNERGY Phase 2 clinical trial [186], which included 299 participants with RRMS and secondary-progressive MS (SPMS), to examine longitudinal changes in myelin and tissue integrity using MRI [188]. SELs were detected through serial T1-weighted and T2-weighted MRI scans, and their progression was assessed using magnetization transfer ratio (MTR) and diffusion tensor imaging (DTI) radial diffusivity (RD), both of which are biomarkers of myelin integrity and axonal damage [188].

The results revealed that SELs exhibited significantly lower MTR values compared to non-SELs, indicating greater demyelination [188]. Additionally, RD values were higher in SELs, reflecting increased tissue damage and axonal degeneration [188]. Over the 72-week study period, SELs continued to show greater declines in MTR and further increases in RD, confirming that these lesions undergo ongoing demyelination and structural deterioration [186]. The correlation between these changes suggests that SELs contribute significantly to the neuropathology of MS, particularly in progressive forms of the disease [186]. Furthermore, while patients with SPMS had more SELs and greater baseline tissue damage than those with RRMS, the rate of change in lesion integrity over time was similar in both groups, indicating that SEL-driven demyelination is an important factor in both relapsing and progressive MS [186].

These findings have profound implications for remyelination therapies as a treatment option for MS. The fact that SELs continue to expand and accumulate damage over time suggests that remyelination therapies may be insufficient if they do not simultaneously address the underlying mechanisms driving lesion progression [186]. The lack of spontaneous remyelination in SELs implies that the lesion microenvironment may be inherently hostile to repair processes, necessitating adjunctive treatments that target chronic inflammation, microglial activation, and metabolic dysfunction within these lesions [186]. Moreover, SELs could serve as valuable imaging biomarkers for monitoring disease progression and evaluating the effectiveness of remyelination therapies [186]. If future treatments can reduce SEL expansion or improve MTR and RD measures, they may provide meaningful clinical benefits for patients with MS [186]. Overall, this study underscores the need for therapies that not only promote remyelination but also mitigate the chronic inflammatory and neurodegenerative processes that drive progressive disability in MS [186].

### 8.6. AFFINITY Study [189]: Phase II randomized, double-blind, placebo-controlled trial evaluating BIIB033 (anti-LINGO-1) as an add-on to disease-modifying therapy in relapsing MS

The AFFINITY trial was a Phase II, multicenter, randomized, double-blind, placebo-controlled study designed to evaluate the efficacy and safety of Opicinumab (BIIB033), a human monoclonal antibody targeting LINGO-1, in individuals with relapsing multiple sclerosis (RMS) [189]. The trial was conceived as a follow-up to the earlier RENEW and SYNERGY studies, which had shown some preliminary evidence that LINGO-1 inhibition could enhance remyelination and neurorepair [183],[186].

In AFFINITY, participants with RRMS were randomized to receive BIIB033 or placebo as an add-on to their existing DMTs, such as interferons or glatiramer acetate [189]. The primary objective was to determine whether BIIB033 could improve functional outcomes and support neural repair beyond what was achievable with standard immunomodulatory therapy alone. Outcome measures included changes in confirmed disability progression, neuroperformance tests, and imaging-based biomarkers such as MTR, which is sensitive to myelin content [188],[189].

Unfortunately, the AFFINITY trial did not meet its primary endpoint, and BIIB033 failed to demonstrate a statistically significant improvement in clinical, or imaging outcomes compared to placebo [189]. The lack of efficacy was consistent across subgroups, including those with shorter disease duration or lower disability at baseline. These results suggest that blocking LINGO-1 alone may not be sufficient to promote meaningful remyelination or functional recovery in patients with more advanced or chronic MS [189].

Despite the disappointing results, the AFFINITY trial contributed valuable insights into the challenges of translating preclinical remyelination strategies into human therapies. Specifically, it highlighted the potential limitations of monotherapy targeting a single pathway, especially in a complex disease like MS where multiple cellular and molecular mechanisms are involved in remyelination failure [107]. Furthermore, the trial reinforced the idea that timing of intervention may be crucial, as remyelination-promoting agents might be more effective in the early stages of demyelination when OPCs are still viable and responsive [160],[162].

In summary, while the AFFINITY trial did not lead to regulatory approval of Opicinumab, it underscores the need for combination approaches, more precise patient stratification, and early intervention to realize the promise of remyelination-based therapies in MS [114].

## 9. Conclusions

In conclusion, we highlight the complex pathophysiology of MS and emphasize the critical importance of remyelination in the treatment of this debilitating disease. MS is characterized by demyelination and subsequent neural damage, driven by autoimmune responses targeting myelin and ODs [190],[191]. The review underscores the significance of targeting remyelination processes, as successful remyelination can protect axons, restore neural function, and potentially halt disease progression [192].

Emerging therapies, such as high-dose supplementation with vitamin D and glutathione, appear effective in regulating immune activity and lowering oxidative burden, thereby supporting remyelination and neuroprotection [27],[28],[124]. Preclinical studies using toxin-induced models of MS have provided valuable insights into the mechanisms of remyelination and the potential of novel therapeutic targets like LINGO-1 antagonists [132]. Clinical trials, such as those investigating BIIB033, have shown encouraging results in enhancing remyelination and improving clinical outcomes in MS patients [179],[180].

The integration of these innovative approaches into clinical practice could revolutionize the management of MS, shifting the focus from merely managing symptoms to promoting CNS repair and long-term recovery. Continued research into the molecular mechanisms underlying remyelination and the development of targeted therapies is essential for advancing MS treatment and improving the quality of life for those affected by this chronic condition.

## 10. Future research directions in remyelination and MS therapy

Despite decades of therapeutic advancement in MS, treatments primarily aim to suppress inflammation than repair damage or restore function. This gap in therapeutic strategy underscores the core motivation behind this paper: to advocate for remyelination as the next frontier in MS management [107]. Unlike immunosuppressive therapies, which merely slow progression, remyelination therapies target the root cause of long-term disability, the loss of myelin and subsequent axonal degeneration [114]. Therefore, shifting the treatment paradigm from immunomodulation to regeneration represents a transformative opportunity for patient care [113].

The failure of recent remyelination-targeted trials, such as AFFINITY, which investigated the efficacy of opicinumab (a LINGO-1 antagonist), highlights the complexity of translating regenerative strategies into clinical success [189]. While initial studies like RENEW and SYNERGY showed hints of promise, AFFINITY failed to meet its primary endpoints, reminding us that therapeutic timing, patient selection, and disease stage are all critical to success [189]. These outcomes reinforce the need for better stratification of trial participants and identification of patients in whom remyelination is biologically feasible [193].

A key area for future development is the creation of reliable, non-invasive biomarkers for remyelination. MRI lacks the resolution to distinguish remyelination and remyelinated axons accurately [188]. Emerging technologies such as myelin water imaging and PET-based tracers are promising tools, but they require validation in larger, longitudinal studies [194]. Incorporating these tools into clinical trials would enable more precise measurement of therapeutic outcomes and accelerate drug development pipelines.

Another promising avenue is the use of combination therapies that target multiple pathways simultaneously. For example, agents like clemastine that stimulate OPC differentiation could be paired with metabolic modulators such as vitamin D or GSH to enhance the CNS microenvironment and support remyelination [109],[125],[195]. This integrative approach acknowledges that remyelination is not a single-step process, but a cascade of cellular and molecular events requiring both intrinsic and extrinsic support [196].

Furthermore, animal models of MS must evolve to better mimic the chronic, progressive nature of human disease. While the cuprizone model is valuable for studying acute demyelination and spontaneous remyelination, it fails to capture the immune complexity and remyelination failure observed in SPMS [134]. Refining or combining models, such as using aged animals or integrating EAE with metabolic stressors, may yield more translatable results [196],[197].

Precision medicine also represents a critical step forward. Stratifying patients based on molecular, genetic, or immunological biomarkers could dramatically improve clinical trial outcomes by identifying responders to remyelination therapies [198]. The future of MS treatment lies not in one-size-fits-all solutions but in personalized interventions that reflect the biological heterogeneity of the disease.

In summary, this paper emphasizes the urgent need to prioritize remyelination in MS therapeutic strategies. By focusing on regeneration rather than suppression, future therapies can move beyond temporary disease control and offer lasting functional recovery. Bridging the gap between promising preclinical insights and clinical reality requires innovative trial designs, better biomarkers, refined animal models, and a commitment to combination strategies. Remyelination is not merely a therapeutic goal, it is a paradigm shift in how we understand and treat MS.

## Use of AI tools declaration

The authors declare they have not used Artificial Intelligence (AI) tools in the creation of this article.
